# Forgotten underwater forests: The key role of fucoids on Australian temperate reefs

**DOI:** 10.1002/ece3.3279

**Published:** 2017-09-10

**Authors:** Melinda A. Coleman, Thomas Wernberg

**Affiliations:** ^1^ NSW Fisheries Coffs Harbour NSW Australia; ^2^ National Marine Science Centre Southern Cross University Coffs Harbour NSW Australia; ^3^ UWA Oceans Institute and School of Biological Sciences University of Western Australia Crawley WA Australia

**Keywords:** climate change, decline, *Ecklonia radiata*, ecology, kelp, seaweed

## Abstract

Kelp forests dominated by species of Laminariales are globally recognized as key habitats on subtidal temperate rocky reefs. Forests characterized by fucalean seaweed, in contrast, receive relatively less attention despite being abundant, ubiquitous, and ecologically important. Here, we review information on subtidal fucalean taxa of Australia's Great Southern Reef, with a focus on the three most abundant and widely distributed genera (*Phyllospora, Scytothalia*, and *Sargassum*) to reveal the functionally unique role of fucoids in temperate reef ecology. Fucalean species span the entire temperate coastline of Australia (~71,000 km^2^) and play an important role in supporting subtidal temperate biodiversity and economic values on rocky reefs as well as in adjacent habitats. Climatic and anthropogenic stressors have precipitated significant range retractions and declines in many fucoids, with critical implications for associated assemblages. Such losses are persistent and unlikely to be reversed naturally due to the life history of these species and colonization of competitors and grazers following loss. Active restoration is proving successful in bringing back some fucoid species (*Phyllospora comosa*) lost from urban shores and will complement other passive and active forms of conservation. Fucalean forests play a unique role on subtidal temperate reefs globally, especially in Australia, but are comparatively understudied. Addressing this knowledge gap will be critical for understanding, predicting, and mitigating extant and future loss of these underwater forests and the valuable ecosystem services they support.

## 1 INTRODUCTION

Underwater macroalgal forests are globally recognized as key components of temperate rocky reefs where they play a disproportionately important role in supporting immense economic and ecologic value (Bennett et al., 2016; Steneck & Johnson, 2013; Steneck et al., 2002). Canopy‐forming macroalgae are considered “foundation species” sensu (Dayton, 1972) that modify environmental conditions and underpin biodiversity both locally (Coleman, Vytopil, Goodsell, Gillanders, & Connell, 2007; Graham, 2004; Irving, Connell, & Gillanders, 2004; Wernberg, Kendrick, & Phillips, 2003; Wernberg, Kendrick, & Toohey, 2005) and in adjacent habitats (Bishop, Coleman, & Kelaher, 2010; Krumhansl & Scheibling, 2012; Vanderklift & Wernberg, 2008). Although these underwater forests often comprise taxonomically diverse canopies of macroalgae from many orders, scientific attention predominately centers on species of Laminariales or true kelps (e.g., *Macrocystis*,* Ecklonia*,* Laminaria,* see however [Fraser, 2012; Bolton, 2016] for a discussion of the definition of “kelp”). This is despite macroalgal species in other orders, especially the Fucales, dominating many underwater forests globally (Steneck & Johnson, 2013; Thibault, Pinedo, Torras, & Ballesteros, 2005; Verges et al., 2014; Vogt & Schramm, 1991; Wernberg, Thomsen, Staehr, & Pedersen, 2004; Wernberg, Kendrick, et al., 2003; Wikström & Kautsky, 2007). Although the similar structural arrangement of laminarian and fucalean forests are often justification for their grouping in temperate reef ecology (Steneck & Johnson, 2013), inherent differences in the biology and morphology of these orders are likely to result in specific biotic associations and responses to environmental change (Hirst, 2006; Phillips, Kendrick, & Lavery, 1997; Wernberg & Connell, 2008; Wernberg, de Bettignies, Bijo, & Finnegan, 2016; Wernberg, Russell, et al., 2011). Hence, addressing the knowledge gap surrounding the specific role of fucoids on subtidal temperate reefs will be critical for understanding how these unique forests and their associated biodiversity will respond to increasing anthropogenic stressors and in informing management and conservation initiatives.

The temperate rocky reefs of the southern hemisphere are unique in that they are home to the greatest diversity of fucoid genera globally supporting 73% more species than reefs in the northern hemisphere (Steneck & Johnson, 2013). Indeed, fucoids are 6 times more speciose than laminariales in the southern hemisphere (Guiry, 2012; Steneck & Johnson, 2013). The microtidal nature of many temperate coasts in Australia means that these fucoids are predominately subtidal, a similar situation to rocky reefs of the Baltic and Mediterranean Seas. In particular, the underwater macroalgal forests that dominate more than 71,000 km^2^ of the temperate Australian coastline (the Great Southern Reef or GSR (Bennett et al., 2016) have the highest fucoid diversity and endemism globally. These forests comprise a diverse flora of 63 species of Fucales (e.g., *Phyllospora comosa*,* Scytothalia dorycarpa, Cystophora* spp., *Cystosiera* spp., *Acrocarpia* spp., *Durvillaea potatorum*, and *Sargassum* spp.) with only four native species of Laminariales (e.g., *Ecklonia radiata, Macrocystis pyrifera, M. angustifolia*, and *Lessonia corrugata*) occurring as either mixed or monospecific forests (Goodsell, Fowler‐Walker, Gillanders, & Connell, 2004; Turner & Cheshire, 2003; Wernberg & Connell, 2008; Wernberg, Thomsen, Tuya, & Kendrick, 2011; Wernberg, Kendrick, et al., 2003). Indeed, mixed laminariales/fucoid forests have been estimated to account for up to 64% of subtidal macroalgal forests in southern and Western Australia (Goodsell et al., 2004; Wernberg, Thomsen, et al., 2011). In particular, extensive monospecific fucoid forests comprised of *Phyllospora comosa*,* Scytothalia dorycarpa* (hereafter referred to as *Phyllospora* and *Scytothalia*, respectively) and species of *Sargassum* occur along most of the ~8,000 km temperate coast of Australia, but have only recently been the focus of research efforts spurred by climatic‐ and anthropogenic‐induced declines (Coleman, Kelaher, Steinberg, & Millar, 2008; Phillips & Blackshaw, 2011; Smale & Wernberg, 2013; Wernberg, Bennett, et al., 2016).

To assess the knowledge base of subtidal fucoids relative to laminariales, we did a literature search (see Appendix S1 for detailed protocol) to qualitatively identify scientific publications on the most abundant subtidal habitat‐forming species in Australian waters. We searched for the terms *Phyllospora comosa*,* Scytothalia dorycarpa, Durvillaea potatorum, Acrocarpia* spp., *Cystophora* spp., *Cystoseira trinodis, Sargassum* spp., *Macrocystis* spp., *L. corrugata*, or *E. radiata* in the title, keywords, or abstract of papers and found between 1 and 25 relevant publications for each fucoid species versus 321 relevant papers on the 3 laminarian taxa, highlighting the critical lack of specific ecological studies on fucoids relative to co‐occurring laminariales. Moreover, this literature search revealed that, relative to laminariales, these fucoids have only been the focus of recent research attention with 80% of all publications on *Scytothalia* and all ecological studies on *Phyllospora* from the last decade. Given the relative lack of studies to conduct a rigorous quantitative review of the importance of subtidal fucoids, here we review knowledge on Australia's extensive subtidal fucoid forests to qualitatively demonstrate their unique role in reef ecology, and identify research gaps that should be addressed to better understand these key habitats and inform conservation and rehabilitation efforts. We specifically focus on the better studied taxa, *Phyllospora;* 25 papers, *Sargassum* spp.; 22 papers, and *Scytothalia*; 8 papers because these are the most dominant habitat‐forming fucoids on the temperate subtidal reefs across Australia and all 3 taxa have undergone recent declines (Coleman, Kelaher, et al., 2008; Phillips & Blackshaw, 2011; Smale & Wernberg, 2013; Wernberg, Bennett, et al., 2016).

## 2 BIOLOGY OF AUSTRALIA'S SUBTIDAL FUCOID FORESTS

### 2.1 Distribution and abundance

**Figure 1:**
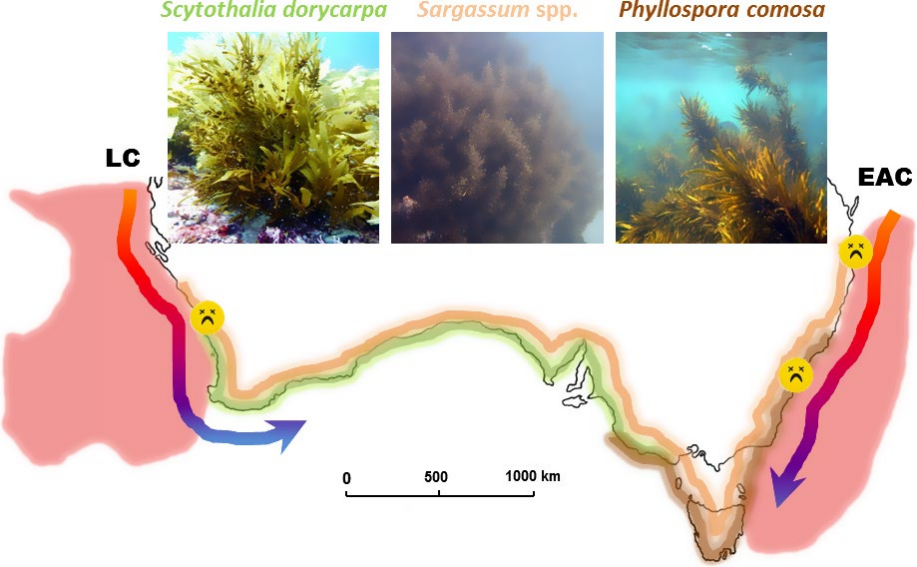
*Phyllospora comosa* (brown), *Scytothalia dorycarpa* (green), and a representative temperate *Sargassum* (*S. linearifolium*; orange) are distributed around the temperate coastline of Australia. These species are dominant or co‐dominant habitat formers with the kelp *Ecklonia radiata* in the east and west, respectively. Major boundary currents shown on the east (East Australian Current; EAC) and west (Leeuwin Current; LC) coasts as well as regions of documented local extirpation (dead smiley). The ocean areas highlighted in red are global warming hotspots, where recent temperature increases have been in the top 10% globally (after Hobday and Pecl 2014)

*Phyllospora comosa* (C. Agardh) and *S. dorycarpa* (Greville) are closely related, monotypic species in the family Seirococcaceae (Huisman, 2000; Womersley, 1987). It is hypothesized that these Tethyan taxa speciated following historically stable, temperate conditions and a lack of mass extinction events along the southern coastline of Australia (Phillips, 2001). They largely occupy allopatric distributions from approximately ~31°S on the east (Coleman, Kelaher, et al., 2008) and west (Smale & Wernberg, 2013) coastlines of Australia, respectively, with only a narrow overlap between Robe (~37°S, South Australia) and Point Lonsdale (~38°S, Victoria; Figure 1). Both species are perennial and common on exposed rocky reefs with *Phyllospora* dominating in shallow (~0–3 m) areas in NSW and deeper (0–10 m) in Tasmania and Victoria (Edgar, 1984; James, Reid, Bone, Levings, & Malcolm, 2013). *Scytothalia* has a wider depth distribution occurring between 3 and 50 m depth (Shepherd & Womersley, 1971; Smale et al., 2010). Both species are canopy forming with *Phyllospora* growing to about 3 m height and *Scytothalia* typically about 1–2 m in height (Huisman, 2000).

In contrast, *Sargassum* is the most speciose genus of fucoids and comprises both temperate and tropical species. There are approximately 19 species of temperate *Sargassum*, many of which span the entire temperate coastline of Australia (e.g., *S. linearifolium*; Figure 1) and a broad depth range. *Sargassum* spp. are characterized by highly variable morphology making species identification difficult and much ecological research on *Sargassum* focuses on the genus level. Unlike *Phyllospora* and *Scytothalia*,* Sargassum* generally forms a mixed subsurface canopy with plants growing to between 10 cm and 2 m in length (Womersley, 1987). Similarly, in contrast to most other fucoids, *Sargassum* spp. are pseudo‐perennial, implying they periodically shed their foliose canopy, only leaving behind the holdfast and short, basal fronds, which may account for highly variable estimates of percentage cover between seasons and over large spatial scales (Bennett & Wernberg, 2014; Thomsen, Wernberg, Staehr, & Pedersen, 2006; Thomson, Babcock, Vanderklift, Symonds, & Gunson, 2012).

The subtidal temperate reefs of Australia are unique in that fucoid forests often form extensive monospecific forests or are co‐dominant in mixed forests with Laminariales (Steneck & Johnson, 2013). *Phyllospora* and *Scytothalia* both occur as monospecific and mixed algal forests, but the distribution of these forest types varies spatially. *Phyllospora* commonly occurs as monospecific forests in shallow, exposed areas (e.g., in NSW) or as mixed forests with *Ecklonia radiata* at its deeper edges (e.g., in Tasmania). Densities of *Phyllospora* are approximately 14 individuals per m^2^ in shallow monospecific forests (Peters, 2015) and 2.5 individuals per m^2^ in deeper mixed forests (Valentine & Johnson, 2004). *Scytothalia* also occurs as either mixed or monospecific forests in its central and southern range (Baker & Edyvane, 2003; Turner & Cheshire, 2003), but occurs as predominately mixed forests at higher latitudes in Western Australia where it is gradually replaced by warmer water Fucales such as *Sargassum* (Wernberg, Thomsen, et al., 2011). Percent cover of *Scytothalia* varies between 2% and 39% throughout its distribution (Bennett & Wernberg, 2014; Smale & Wernberg, 2013) and tends to increase with latitude (Smale et al., 2010; Wernberg, Thomsen, et al., 2011) and wave exposure (Turner & Cheshire, 2003; Wernberg & Connell, 2008). Relative to *Ecklonia*,* Scytothalia* also dominates on high relief reefs and on granite compared to limestone (Harman, Harvey, & Kendrick, 2003). Regardless, whether occurring as mixed or monospecific forests, fucoids and laminariales are integrally linked on the subtidal temperate reefs of Australia, but scientific knowledge is biased toward the latter.

*Sargassum* tends to form mixed stands with other fucoids and laminariales and often up to nine species of *Sargassum* co‐occur within the same stand (Goldberg, 2007). Accordingly, percentage cover of *Sargassum* in Western Australia varies between 0% and 20%, with the remaining reef cover predominately being composed of *Scytothallia* (0%–40%) and *Ecklonia* (40%–86%; Bennett & Wernberg, 2014). The relative cover of *Sargassum* spp. changes on small spatial scales but has been shown to have the greatest cover at mid‐latitudes (~30.5°) and be correlated with temperature (Wernberg, Thomsen, et al., 2011).

### 2.2 Morphology and life history

**Figure 2:**
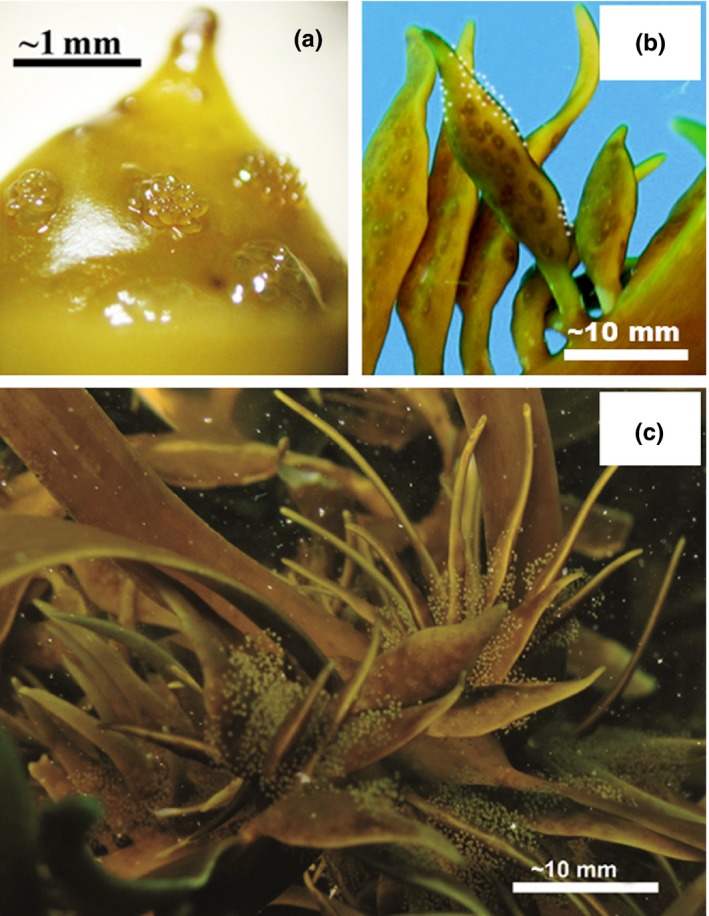
Photographs showing key aspects of *Scytothalia* morphology and life history. (a) Egg release from conceptacles. (b) Specialized receptacles (bearing multiple conceptacles) are produced on the main axis. (c) Gametes are retained in a cloud of mucous during synchronous gamete release in the field. Photos: Stefan Andrews

*Phyllospora* and *Scytothalia* are morphologically similar with flattened thalli (Figure 1, (Womersley, 1987)) and a holdfast with densely packed haptera. *Phyllopora* has a flattened main axis which bears many closely set lateral branches and has vesicles and a short stipe. In contrast, *Scytothalia* has flattened alternate axes. Reproductive structures (conceptacles) are produced on the entire surface of lateral branches in *Phyllospora,* whereas *Scytothalia* produces special receptacles arising from the lateral branches (Figure 2). *Sargassum* spp. have varied morphology, but are generally foliose and bushy with reproductive upper parts of the thallus morphologically distinct from the lower perennial portions of the plant which have wider basal fronds (Figure 1). *Phyllospora* and *Sargassum* both have gas‐filled vesicles which assist it to be positioned above the substratum when attached and float on the surface of the water when detached following storms (Figure 2). Indeed, the presence of gas‐filled vesicles facilitates long distance dispersal relative to species that lack such structures (Coleman, Chambers, et al., 2011). The morphology of all three of these fucoid taxa contrasts starkly with all co‐occurring species of laminariales which, in comparison, have structurally simple thalli.

There is little published knowledge on spatial variation in morphology in each fucoid taxa, but the data that do exist demonstrate great spatial variation and suggest that local environmental conditions may dictate morphology as is known for other macroalgae (Wernberg, Coleman, Fairhead, Miller, & Thomsen, 2003). For example, stipe length in *Phyllospora* is significantly shorter at lower latitudes in NSW with some low latitude locations (Port Stephens) characterized by plants with significantly more main axes and fewer vesicles (Coleman, unpubl. data). In contrast, others have found *Phyllospora* to be longer at its northern limits (~31°S, Port Macquarie) and stipe length to be greater in southern NSW (Peters, 2015). All other morphologic characteristics of *Phyllospora* exhibit much small‐scale variation along its entire geographic distribution suggesting that morphology may be highly plastic. There are no large‐scale morphologic studies within species of *Sargassum* likely because similarities in morphology among species make single‐species identification challenging. It is thought, however, that local environmental conditions underpin highly plastic morphologies within species. Examining the extent of covariation in individual morphological characteristics among multiple species along environmental gradients may help elucidate what drives this plasticity (Coleman & Muhlin, 2008).

*Phyllospora*,* Scytothalia*, and *Sargassum* spp. have typical fucoid life history strategies producing eggs and sperm which are externally fertilized (Figure 2). *Phyllospora* is dioecious with male individuals releasing motile sperm from elongated conceptacles that fertilize stalked eggs that are released and attached to the female plant (Womersley, 1987). *Phyllospora* sex ratios are approximately 50:50 along much of the coast of NSW (Coleman, unpubl data). Similarly, *Sargassum* can be monoecious or dioecious and many species retain fertilized eggs on the parent plant and zygotes develop for ~24 hr before release (May & Clayton, 1991; Shepherd & Edgar, 2013). In contrast, *Scytothalia* is monoecious with mostly unisexual conceptacles. Reproductive structures appear to be present on both *Phyllospora* and *Scytothalia* throughout the year (Andrews, Bennett, & Wernberg, 2014; Burridge & Hallam, 1993), but *Scytothalia* reproduction may peak in the austral winter (May to September; Andrews et al., 2014). The timing of *Sargassum* reproduction varies greatly among species (Shepherd & Edgar, 2013). There exists little information on what cues reproduction in these taxa but it is likely that, as with other fucoids, release of gametes is cued to environmental conditions including periods of calm water (Andrews et al., 2014; Figure 2), high, slack, or neap tides or lunar cycles (May & Clayton, 1991; Muhlin, Coleman, Rees, & Brawley, 2011; Pearson & Serrão, 2006). For all taxa, gamete release can easily be induced in the laboratory by subjecting individuals to a series of osmotic and hydrostatic shocks making them ideal species of studies of early life history processes.

Despite many fucoid taxa having easily manipulated life histories, there are only a handful of studies that examine factors affecting the early life history stages of *Phyllospora*,* Scytothalia*, and *Sargassum*. Laboratory studies have shown that fertilization, germling survival, and recruitment are all negatively impacted by high temperature in *Scytothalia* (Andrews et al., 2014). Moreover, the ease of inducing reproduction and growing juvenile *Phyllospora* has made this species a model system for marine toxicology studies (Burridge & Bidwell, 2002). Relative to other species, the juvenile stages (zygotes, germlings) of *Phyllospora* are particularly sensitive to a range of contaminants, including sewage effluent, oil dispersants, formaldehyde, and tributyltin with sensitivity decreasing with age (Burridge, Lavery, & Lam, 1995; Burridge, Portelli, & Ashton, 1996; Burridge & Shir, 1995). Studies on other fucoids suggest that warming temperatures may impact early life history via alteration of zygote attachment time (Coleman & Brawley, 2005a) and reproductive timing (Coleman & Brawley, 2005b). Thus, further studies on the early life history stages of both *Phyllospora*,* Scytothalia*, and *Sargassum* will be crucial for understanding the mechanisms behind recent climate‐mediated population declines and failure to naturally re‐establish.

Dispersal of *Phyllospora* and *Sargassum* zygotes appears to occur predominately over short distances as indicated by declining recruitment within distance from adult canopies (Campbell, Marzinelli, Vergés, Coleman, & Steinberg, 2014; Kendrick & Walker, 1991, 1995). Furthermore, genetic studies confirm these observations and reveal that inbreeding in *Phyllospora* is common and gene flow may break down on scales <80 km (Coleman, Dolman, Kelaher, & Steinberg, 2008; Coleman & Kelaher, 2009). Dispersal of *Phyllospora* is also influenced by coastal topography and/or hydrodynamics with populations inhabiting enclosed bays showing significantly greater genetic structuring (Coleman, Chambers, et al., 2011). Over evolutionary scales, low genetic diversity of *Phyllospora* in southeastern Australia suggests that colonization may be recent and rapid dispersal facilitated by the presence of gas‐filled vesicles (Durrant, Barrett, Edgar, Coleman, & Burridge, 2015). Indeed, gas‐filled vesicles facilitate dispersal of *Phyllospora* over at least ~600 km and drift material has been found on Lord Howe Island despite the plants not growing there (Millar & Kraft, 1993). Furthermore, *Phyllospora* is often found washed up on Sydney's beaches and has dispersed in ocean currents from extant sites to the north or south (10s km; (Coleman, Kelaher, et al., 2008)). Certainly, the East Australian Current is likely to facilitate dispersal of this species (Coleman, Roughan, et al., 2011). Dispersal of fertile drift material, however, does not guarantee gene flow. Viable male and female plants need to be present in sufficient density for fertilization to occur and subsequent recruitment is likely mediated by pre‐emptive competition (Coleman, Kelaher, et al., 2008) and extant environmental conditions (Smale & Wernberg, 2013). There are no genetic studies on *Scytothalia*, but the lack of vesicles to facilitate long‐range dispersal as well as ecological experiments indicating a high level of ecotypic differentiation in physiology among populations (Bennett, Wernberg, Joy, De Bettignies, & Campbell, 2015), suggesting dispersal and gene flow are relatively limited, and may be less than *Phyllospora*. Certainly, the presence of gas‐filled vesicles to aid dispersal and maintain gene flow among populations may be particularly advantageous in a future of increasing population fragmentation (Coleman, Kelaher, et al., 2008) and changing oceanic vectors of dispersal (Cetina‐Heredia, Roughan, van Sebille, & Coleman, 2014; Cetina‐Heredia, Roughan, van Sebille, Feng, & Coleman, 2015; Coleman et al., 2017). The lack of such structures combined with the predominately poleward flow of boundary currents may also explain the failure of *Scytothallia* to re‐establish following climate‐mediated loss at its range limits in Western Australia (Wernberg, Bennett, et al., 2016).

Recruitment processes are well studied in *Sargassum* relative to other fucoids. At mid‐latitudes (~32°), the presence of an adult canopy has little influence on recruit survival until recruits are 6 months of age, after which, canopy negatively impacts recruit survival (Kendrick, 1994). Interestingly, this pattern may be reversed at lower (warmer) latitudes where *Sargassum* recruits may rely heavily on the presence of adult canopy for survival, something that also applies to *Scytothalia* recruits (Bennett & Wernberg, 2014). Unlike most other fucoids, *Sargassum* has the ability to regenerate from remnant holdfasts that remain following loss of the thallus, which tends to decrease spatial and temporal variability in adult density (Kendrick & Walker, 1994).

### 2.3 Physiology and growth

In contrast to co‐occurring laminariales (e.g., *E. radiata*), research on the physiology of *Phyllospora*,* Scytothalia*, and *Sargassum* spp. is scant but will be critical to understanding the mechanisms behind recent declines in all three taxa. *Phyllospora* photosynthetic efficiency, growth, and survival appear to be negatively correlated with higher summer temperatures (22°C; Flukes, Wright, & Johnson, 2015) and in NSW, plants have higher δ^13^C, photosynthetic capacity (rETRmax), and concentrations of chl c and fucoxanthin compared to Tasmania. However, as with morphology (Peters, 2015), these traits appear to be highly plastic and rapidly converge under similar environmental conditions (Flukes et al., 2015). Supporting this finding, Weigner (2016) found no latitudinal pattern in *Phyllospora* tissue chemistry along a 5 degree latitudinal gradient in NSW. In contrast to *Phyllospora*,* Scytothalia* and *Sargassum* have lower pigment (Chl a, Chl c) concentrations at lower latitudes, and these tissue changes appear to be part of systematic physiologic changes of increasing respiration and decreasing net photosynthesis at lower latitudes, corresponding to higher water temperatures (Wernberg, de Bettignies, et al., 2016). *Scytothalia* from Perth (~32°S) has its physiologic optimum (highest net primary production) around 24°C (Smale & Wernberg, 2013; Wernberg, de Bettignies, et al., 2016), which is lower than co‐dominant canopy‐forming species including *Ecklonia* and *Sargassum* (Wernberg, de Bettignies, et al., 2016). In addition, *Scytothalia* has a narrower temperature range for optimum performance than these species, being almost half that of *Sargassum* (Wernberg, de Bettignies, et al., 2016). These short‐term physiologic optima are, however, higher than observed for other biological processes. Specifically, recruitment and recruit development (Andrews et al., 2014) and linear growth (Xiao et al., 2015) have been found to decrease and eventually cease at about ~20°C. Importantly, reciprocal transplant experiments have clearly documented that these temperature thresholds vary between populations at different latitudes (Bennett et al., 2015).

## 3 KEY FOUNDATION SPECIES IN SHALLOW SUBTIDAL AREAS

**Figure 3:**
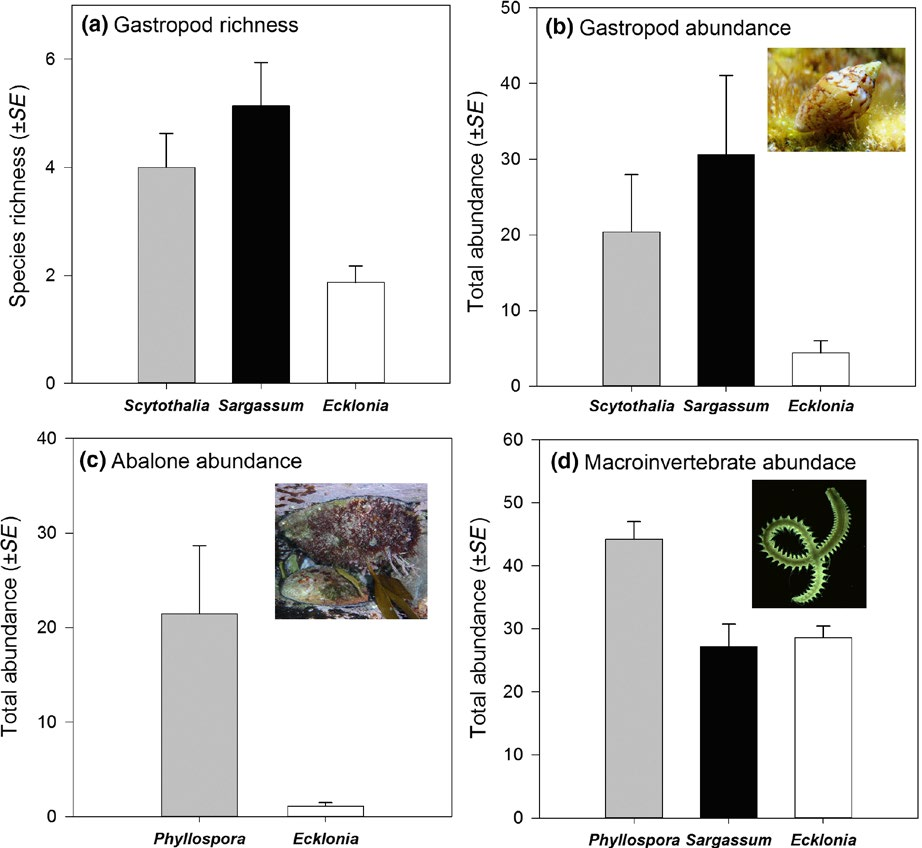
*Phyllospora*,* Scytothalia*, and *Sargassum* spp. support unique and diverse associated assemblages relative to co‐occurring laminariales (*Ecklonia radiata*). *Scytothalia* and *Sargassum* support higher (a) richness and (b) abundance of gastropods (data re‐analyzed from Tuya et al. 2008). *Phyllospora* supports (c) higher abundances of abalone (data re‐analyzed from Marzinelli et al. 2014), and (d) higher total abundance of infauna in adjacent soft‐sediment habitats enriched with detritus from *Ecklonia* and *Sargassum* (data re‐analyzed from Bishop et al. 2010)

Subtidal fucoid forests play a key role on Australia's temperate rocky reefs as foundation species that support biodiversity (Figure 3). Indeed, despite the grouping of fucoids with Laminariales due to their structural similarity as canopy formers (Steneck & Johnson, 2013), we show here that fucoid species play unique functional roles in structuring biodiversity of temperate reefs and should be considered separately, particularly where they occur as dominant monospecific forests (e.g., *Phyllospora* along the east coast of Australia).

*Phyllsopora* and *Scytothalia* occur either as primary constituents of monospecific forests or as components of mixed algal canopies (Goodsell et al., 2004; Irving et al., 2004), whereas *Sargassum* spp. generally occur in mixed stands (Goldberg, 2007; Wernberg & Connell, 2008; Wernberg, Thomsen, et al., 2011). Indeed, even mixed fucalean/laminarian forests have been shown to provide different abiotic environmental conditions in terms of light, abrasion, and sedimentation (Irving & Connell, 2006; Wernberg et al., 2005) and support unique phytal (Tuya, Wernberg, & Thomsen, 2008; Wernberg et al., 2004), benthic (Goodsell et al., 2004; Irving et al., 2004), and fish (Harman et al., 2003; Tuya, Wernberg, & Thomsen, 2009) communities compared to monospecific laminarian forests. Surprisingly, less is known about the key role these taxa play in supporting biodiversity in monospecific forests despite the prevalence of monospecific fucoid forests around the entire temperate coast of Australia (Connell & Irving, 2008; Wernberg, Thomsen, et al., 2011). Certainly, monospecific forests of *Phyllospora* support unique epifaunal (Marzinelli, Leong, Campbell, Steinberg, & Verges, 2016) and understory communities and greater abundances of economically important species such as abalone (Marzinelli et al., 2014; Figure 3) indicating that they play an important functional role in supporting temperate biodiversity. Interestingly, *Phyllospora* did not support statistically different roving fish communities to the co‐dominant *E. radiata*, but it is likely that small and cryptic species may respond to differences in these macrophyte habitats (Marzinelli et al., 2014). No equivalent comparative community‐level studies have been done for *Scytothalia* or *Sargassum*, but studies focused on the gastropod component of epifauna clearly indicate that *Scytothalia* supports different communities compared with the dominant *Ecklonia* (Tuya et al., 2008; Figure 3). Similarly, *Sargassum* spp. can support unique epifaunal communities relative to *Ecklonia* and *Phyllospora* and a greater diversity of epifauna per gram, probably because of its structurally complex form (Marzinelli et al., 2016). Indeed, both natural and experimental losses of *Phyllospora* and *Scytothalia* canopies have been shown to result in significant changes in associated communities (Coleman, Kelaher, et al., 2008; Smale & Wernberg, 2013; Valentine & Johnson, 2004), suggesting that loss and decline of these fucoids will have widespread ecosystem level impacts.

*Sargassum* (particularly *S. linearifolium*) is a habitat for diverse assemblages of mesograzers and has been used as a model system for plant–herbivore interactions (Poore & Hill, 2005; Poore & Steinberg, 1999; Poore et al., 2013). Some temperate *Sargassum* spp. exhibit tolerance to grazing via compensatory growth (Hay et al. 2011) and temperate *Sargassum* spp. characterized by relatively high levels of phenolics are equally as palatable to tropical fishes as phenolic poor tropical *Sargassum* spp. (Steinberg et al. 1991). This may change, however, under future temperature and acidification scenarios. Palatability of *Sargassum* to amphipods was greater under acidified and elevated temperature conditions, suggesting that the nutritional content or changes in algal growth form may alter trophic dynamics under future ocean conditions (Hay et al. 2011).

Forests of *Scytothalia* and *Phyllospora* also play a role in supporting many important near‐shore fisheries and are therefore important economic components of temperate reefs. For example, *Phyllospora* forests support significantly more abalone than other macroalgal habitats (Marzinelli et al., 2014; Figure 3). Interestingly, the loss of *Phyllospora* from the Sydney region may have exacerbated the failure of abalone to re‐establish viable populations in this region following abalone loss through *Perkensis* disease. Furthermore, *Phyllospora,* which is commonly known as “crayweed” is thought to support high densities of adult lobster (pers. obs. MA Coleman) and may also be important in enhancing lobster recruitment and decreasing predation on juveniles (Hinojosa, Green, Gardner, & Jeffs, 2015).

Detached habitat‐forming macroalgae play key roles as spatial subsidies, supporting communities in other habitats after being removed from the seafloor following storms (see refs above). *Phyllospora* and *Sargassum* spp. are particularly important spatial subsidies in adjacent soft‐sediment communities because the presence of gas‐filled vesicles that allow them to float, confer ability to disperse into new and distant areas after being detached. *Phyllospora* is a common component of wrack that washes up in estuarine and beach soft‐sediment communities and sediments containing *Phyllospora* detritus support unique infaunal communities and higher total abundance of infauna relative to other species, including *Sargassum* spp. and *Ecklonia radiata* (Bishop et al., 2010; Figure 3). Similarly, floating *Phyllospora* and *Sargassum* wrack supports transient, yet diverse pelagic fish communities (Dempster & Kingsford, 2004) and presumably acts as a dispersal vehicle for a diverse range of marine organisms as is known from other floating seaweeds (Fraser, Nikula, & Waters, 2011). *Scytothalia* and *Sargassum* also play key roles as spatial subsidies to seagrass communities and reefs several kilometers away, where they are rapidly consumed by fauna including herbivorous fish and sea urchins (Vanderklift & Wernberg, 2008). Fucoids are, therefore, key trophic components in near‐shore food webs.

## 4 THREATS AND DECLINES OF AUSTRALIA'S FUCOID FORESTS

Recent declines, fragmentation, and losses of *Phyllospora*,* Scytothalia*, and *Sargassum* spp. have highlighted the vulnerability of fucoid forests to a range of anthropogenic stressors. Among the most significant losses of fucoid canopies in Australia is the 100 km range retraction of *Scytothalia* from Western Australia following the 2011 marine heatwave (Smale & Wernberg, 2013). During the heatwave, temperatures increased well above the physiologic tolerance of *Scytothalia* for more than 10 weeks. Prior to the heatwave, which also caused a range of other ecological impacts (Pearce et al., 2011; Wernberg, Bennett, et al., 2016), *Scytothalia* was common and highly abundant in Jurien Bay (~30°S). Subsequently, however, it disappeared, contracting south to Wedge Island (Smale & Wernberg, 2013). The loss of *Scytothalia* was associated with substantial changes in habitat and community structure in Jurien Bay (Smale & Wernberg, 2013; Wernberg et al., 2013). A subsequent study has found these changes to correspond to exceedance of the ~2.5°C temperature anomaly (Bennett et al., 2015). Interestingly, this study found populations at the center of their distribution to be equally vulnerable (i.e., to perish under a similar temperature anomaly) as range edge populations, presumably due to little population connectivity and high ecotypic differentiation.

On the east coast of Australia, *Phyllospora* and *Sargassum* spp. have also undergone similarly significant loss and fragmentation around urban areas (Coleman, Kelaher, et al., 2008; Phillips & Blackshaw, 2011). Herbarium specimens and historical photos demonstrate that *Phyllospora* was once abundant along the entire coastline of metropolitan Sydney, but completely disappeared decades ago leaving a 70 km gap in its distribution (Coleman, Kelaher, et al., 2008). Although the exact cause of its decline is unknown, its loss is spatially and temporally correlated to Sydney's former sewage outfalls which discharged large volumes of sewage directly into the near‐shore habitat occupied by *Phyllospora* (Coleman, Kelaher, et al., 2008). This hypothesis is supported by studies demonstrating that *Phyllospora* germlings are more sensitive to sewage effluent than other fucoid species (Burridge et al., 1996). Furthermore, *Phyllospora* disappeared after the installation of a sewage outfall at Ulladulla (May, 1985) and brown algae declined in general with increasing proximity to a sewage outfall in Sydney (Borowitzka, 1972). Although these outfalls have now been moved offshore and water quality in Sydney has greatly improved (Scanes & Phillip, 1995), *Phyllospora* has not returned naturally (Coleman, Kelaher, et al., 2008). Similarly, historic records demonstrate that *Sargassum* spp. have undergone range retractions in southern Queensland, likely due to urbanization (Phillips & Blackshaw, 2011) and failure to recover may be linked to the reliance of adult canopy to ameliorate environmental conditions for recruits (Bennett & Wernberg, 2014) or inability to disperse large distances among rocky headlands (Kendrick & Walker, 1991, 1995). These losses highlight the potential vulnerability of fucoid forests to anthropogenic change and the need for specific monitoring of these key taxa as well as targeted studies to better understand the processes that underpin loss to inform management and conservation initiatives.

Loss and decline of fucalean canopies, regardless of the cause, may itself pose a new threat to marine environments by facilitating proliferation of invasive species. Indeed, both natural and experimental *Phyllospora* canopy removals in Tasmania result in rapid establishment of the invasive kelp, *Undaria pinnitifida* which benefits from available space and increased light following canopy loss (Valentine & Johnson, 2004). Similarly, it is hypothesized that loss of *Phyllospora* in the Sydney region (Coleman, Kelaher, et al., 2008) may have facilitated a local proliferation of the range expanding, *Caulerpa filiformis* which exclusively occupies *Phyllospora's* former habitat (Glasby, Gibson, West, Davies, & Voerman, 2015). Interestingly, expanding beds of *C. filiformis* can then have cascading effects on nearby *Sargassum* beds, negatively influencing photosynthetic condition (Zhang, Glasby, Ralph, & Gribben, 2014) and decreasing the abundance of epifauna (Lanham, Gribben, & Poore, 2015).

An emerging threat to the long‐term persistence of macroalgal forests is the reduction in genetic diversity caused by increased habitat fragmentation and reduced connectivity. Genetic diversity confers adaptive capacity to populations and may allow them to persist through changing environmental conditions (Hughes, Inouye, Johnson, Underwood, & Vellend, 2008; Reusch, Ehlers, Hämmerli, & Worm, 2005; Wernberg et al., in review). Reduced diversity can limit the ability of a population to respond to stressors because fewer genotypes means a limited range of physiologic responses are available to cope with change (Wernberg et al., unpbl. manuscript). This scenario may arise in populations of *Phyllospora* and *Scytothalia* that are isolated either by natural (Coleman, Chambers, et al., 2011; Coleman, Roughan, et al., 2011) or anthropogenic (Coleman, Kelaher, et al., 2008) fragmentation. Indeed, connectivity in macroalgae predicts population persistence with population extirpation being inversely correlated with connectivity (Castorani et al., 2015). Thus, loss and fragmentation of fucoid forests can increase the vulnerability of remaining populations because connectivity is often eroded.

The actual physiologic or ecological reasons behind loss of fucoid forests are often unknown with losses usually correlated to changes in environmental conditions. This is primarily because losses are often documented post hoc and early warning signs that may hint at the process responsible are missed or unknown. For example, loss of *Phyllospora* from the Sydney region was indirectly correlated with sewage effluent, but it is likely that this sewage directly impacted *Phyllospora* germling survival (Burridge et al., 1996) or may have facilitated disease (Campbell, Verges, & Steinberg, 2014; Peters, 2015). Similarly, the range retraction of *Scytothalia,* while correlated with temperature (Smale & Wernberg, 2013), was likely a direct result of physiologic stress killing adult thalli (Smale & Wernberg, 2013; Xiao et al., 2015) coupled with reproduction and recruitment failure in subsequent warm years (Andrews et al., 2014; Bennett et al., 2015). However, there is some evidence that fucoid physiology may be highly plastic and somewhat able to cope with stressors. It has been experimentally demonstrated that *Scytothalia* can acclimate in response to UVB and increased its light absorption efficiency in the UV bands by upregulating synthesis of photoprotective compounds (Xiao et al., 2015). Similarly, *Phyllospora* physiology is also highly plastic and rapidly responds to change in abiotic conditions (Flukes et al., 2015). Certainly, thresholds exist beyond which these species will be unable to compensate, and these thresholds are likely to be determined by a range of factors including dispersal capacity and population connectivity mediating genetic diversity and capacity to adapt (Wernberg et al., in review). Identifying where these thresholds lie may allow better understanding of when and where significant loss of subtidal Fucoid forests may occur. Determining the direct processes behind loss of these important forests will be key for early detection of loss and, where possible, managing the processes responsible.

## 5 THE FUTURE OF AUSTRALIA'S SUBTIDAL FUCOID FORESTS

**Figure 4:**
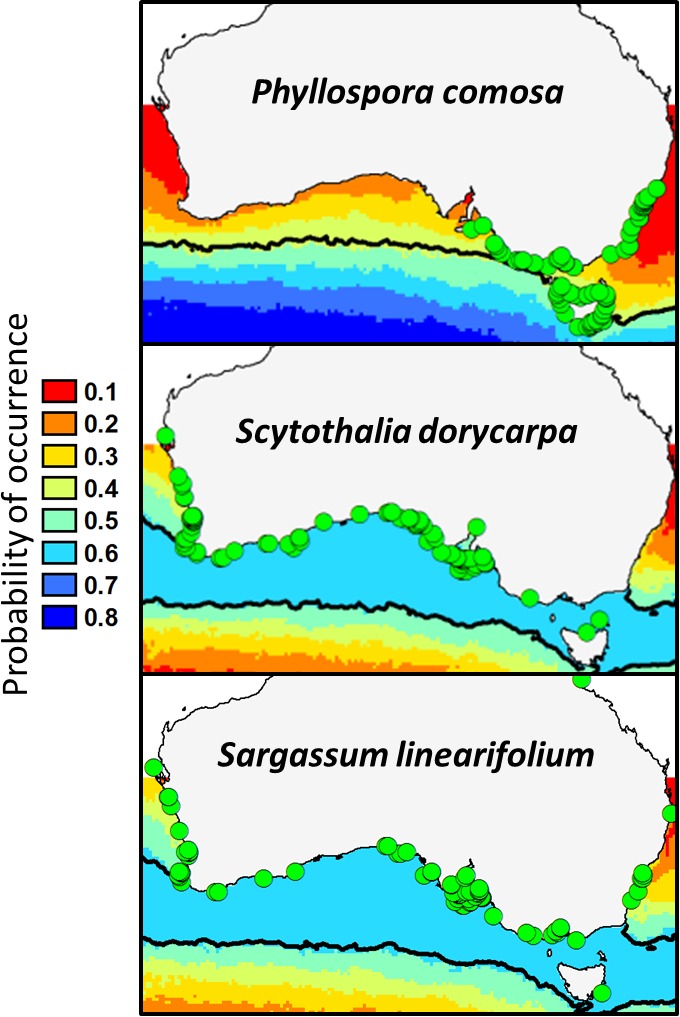
Future distribution of *Phyllospora*,* Scytothalia*, and *Sargassum linearifolium* based on species distribution models under the A1B scenario of ocean warming for 2100. The green dots represent confirmed presences (by 2009) as vouched herbarium specimens. Ocean color represents the probability of occurrence in year 2100. Presence is interpreted as *p* > .5 (black lines; green and blue colors). Data from Martínez et al. (unpbl. manuscript)

Given the strong relationship between temperature and performance of *Phyllospora, Scytothalia*, and *Sargassum* spp. (see above), species distribution models project substantial future declines in all three taxa as a consequence of ocean warming (Figure 4; Martínez et al., unpbl. manuscript). These models suggest that all three fucoids will retreat toward higher latitudes. Projections for 2100 based on the intermediate A1B carbon emission scenario suggest that *Phyllospora* would be restricted to southwestern Tasmania and would lose 74.6% of its current distribution (Figure 4; Table 1). *Scytothalia*, in contrast, would “only” lose 18.6% of its current distribution along the west coast, whereas populations along the south would remain intact (Figure 4). *Sargassum linearifolium*, one of the most widespread temperate fucoids in Australia, would lose 24.3% of its current distribution mainly from its northeastern and northwestern margins (Figure 4; Table 1). The relatively low projected habitat loss by 2100 for *Scytothalia* and *S. linearifolium* are due to their extensive south coast distribution which would remain intact. However, both species would be compressed into a narrow latitudinal range, on the poleward facing edge of the continent where both species would be vulnerable to rapid habitat loss over an extensive area with additional warming.

**Table 1:** Extent of current and predicted future (2100) distribution for *Phyllospora comosa*,* Scytothalia dorycarpa*, and *Sargassum linearifolium* in temperate Australia

Species	Current distribution (km coastline)	Future distribution (km coastline)	Predicted habitat loss (km coastline)	Predicted habitat loss (% of current distribution)
*Phyllospora comosa*	5,547	1,409	4,138	74.6
*Scytothalia dorycarpa*	10,300	8,389	1,911	18.6
*Sargassum linearifolium*	11,844	8,969	2,874	24.3

Total length of temperate coastline was estimated to be 26,730 km. Data from Martínez et al. (unpbl. manuscript).

Predicted (Figure 4) and extant losses of *Phyllospora*,* Scytothalia*, and *Sargassum* spp. (Coleman, Kelaher, et al., 2008; Phillips & Blackshaw, 2011; Smale & Wernberg, 2013; Wernberg, Bennett, et al., 2016) as well as declines in other subtidal fucoid forests globally (Airoldi & Beck, 2007; Thibault et al., 2005; Vogt & Schramm, 1991) suggest that management intervention will be critical to the long‐term persistence of these key temperate habitats. This could involve passive (habitat protection), active (e.g., restoration), anticipatory (e.g., selective breeding or assisted adaptation) or integrated initiatives to halt further loss, boost the resilience of existing forests or restore areas that have already suffered decline.

Passive approaches have traditionally been employed for managing subtidal macroalgal habitats and include the establishment of MPAs to remove the direct and indirect effects of harvesting pressure. The establishment of MPAs and associated trophic structures has been successful in restoring kelp (Laminariales) habitats to many areas globally, for example, see review by Babcock et al. (2010), but the extent to which this effect extends to fucoids is largely unknown because these taxa are often not sampled separately from Laminariales and are simply included within a “canopy‐forming” or “kelp” category. There is some evidence for an increase in shallow mixed fucoid canopies in MPAs following 24 years of protection in New Zealand (Babcock, Kelly, Shears, Walker, & Willis, 1999). Similarly, Barrett, Buxton, and Edgar (2009) found some species of fucoids (*Acrocarpia*) to exhibit more stability within an MPA than fished areas following 10 years of protection, but this pattern was not spatially general. Preliminary sampling of shallow fucoid forests in NSW MPAs indicates little change in *Phyllospora* and *Sargassum* abundances after 8 years of protection (Coleman, Palmer‐Brodie, & Kelaher, 2013). Although it is likely that this and many other Australian MPAs are still too young for restoration of trophic linkages to be fully realized (Babcock et al., 1999), the experimental demonstration that removal of grazing pressure (urchins) results in an increase in *Phyllospora* (Ling, 2008) warrants specific inclusion of fucoids into MPA sampling programs, especially in places where monospecific forests play a key role in temperate ecosystems (e.g., NSW).

**Figure 5:**
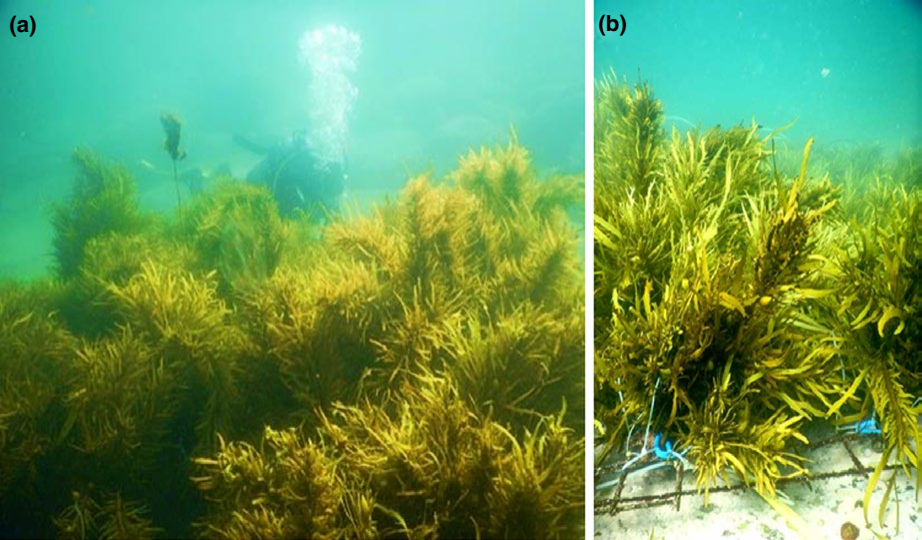
Restoration of *Phyllospora* into areas where it was lost in Sydney. Panels showing (a) diver near a 5 × 5 m restored patch and (b) donor plants attached to mesh with rubber tubing. Photos: E. Marzinelli

Lost fucoid forests may be unable to return to their former state even if favorable environmental conditions are restored, for example, see review by Filbee‐Dexter and Scheibling (2014). Thus, where regime shifts have occurred and passive management (e.g., MPAs) has not resulted in recovery of lost habitats, active intervention may be required. For example, complete loss of *Phyllospora* from the Sydney region (Coleman, Kelaher, et al., 2008) and its inability to re‐establish after decades despite improvements in water quality (Scanes & Phillip, 1995) and adequate dispersal potential (Coleman & Kelaher, 2009) has demonstrated that active, as opposed to passive management action is required to restore these forests. This has prompted government‐funded restoration initiatives (http://www.marine.nsw.gov.au/data/assets/pdf_file/0009/595044/hawkesbury-shelf-discussion-paper.pdf) that are backed by extensive research demonstrating *Phyllospora* is not functionally redundant and warrants restoration (Marzinelli et al., 2014, 2016) and that optimized restoration techniques are successful (Campbell, Marzinelli, et al., 2014). Indeed, initial restoration efforts demonstrated that restored *Phyllospora* populations rapidly become self‐sustaining even if transplanted individuals were lost (Campbell, Marzinelli, et al., 2014; Figure 5), thus overcoming a major impediment to many marine macrophyte restoration efforts. Studies on *Scytothalia* and *Sargassum*, however, suggest that facilitation by an adult canopy maybe critical for successful recruitment, particularly at low latitudes (Bennett & Wernberg, 2014) and successful restoration techniques for these species may necessitate the maintenance of donor plants. Regardless, fucoid algae may be ideal candidates for restoration due to their life history whereby reproduction can easily be induced during restoration activities, prior to transplanted individuals potentially being lost due to storms or grazing.

Integrated approaches to management of marine environments that involve both active and passive initiatives are likely to be most successful in a future of increasing stress. Such approaches acknowledge that we need to not only improve abiotic conditions (e.g., improve water quality) or limit anthropogenic impacts (e.g., MPAs), but it will often be necessary to concurrently actively intervene to restore lost habitat or even anticipate future loss. Integrated approaches to marine management that focus on fucoid forests are currently being investigated in NSW (http://www.marine.nsw.gov.au/key-initiatives/threat-and-risk-assessment-framework). The Mediterranean, with its long history of anthropogenic use, is a classic example of where such integrated approaches will be required to ensure the long‐term persistence of subtidal fucoid forests. In this case, MPAs alone are largely ineffective in restoring underwater fucoid (*Cystoseira*) forests (Mangialajo et al., 2008) that were lost due to processes including overgrazing (Vergés et al., 2014), beach nourishment, storms, habitat loss, and poor water quality (Perkol‐Finkel & Airoldi, 2010). Active intervention (restoration) in combination with improvement in water quality, and MPAs to facilitate dispersal, are suggested to be an effective strategy to bring back these lost forests to the Mediterranean (Airoldi & Beck, 2007; Gianni et al., 2013; Mangialajo et al., 2008). Anticipatory approaches such as selective breeding or assisted adaptation (Aitken & Whitlock, 2013), although potentially controversial, may also provide avenues for boosting the resilience of macroalgal populations against future change and ensure the long‐term persistence of these critical habitats.

## 6 CONCLUSION

Despite being a unique and important component of temperate reefs in Australia, subtidal fucoid forests have been understudied relative to their laminarian counterparts accounting for ~20% of the scientific literature. In Australia, these neglected forests cover over half the continent (~8,000 km coastline, [Bennett et al., 2016]) and play a key role in supporting temperate biodiversity (Bishop et al., 2010; Irving et al., 2004; Marzinelli et al., 2014). Fucoid forests play similarly important roles on subtidal rocky reefs in many other parts of the world (Schiel, 1988; Tanaka, Taino, Haraguchi, Prendergast, & Hiraoka, 2012; Thibault et al., 2005; Wikström & Kautsky, 2007) but are rarely studied in their own right, except where these forests are the only habitat formers (i.e., monospecific forests of *Cystosiera* in the Mediterranean or *Fucus* in the Baltic).

Natural and anthropogenic stressors have precipitated recent large‐scale declines in subtidal fucoid forests globally (Coleman, Kelaher, et al., 2008; Nilsson, Engkvist, & Persson, 2004; Phillips & Blackshaw, 2011; Smale & Wernberg, 2013; Tanaka et al., 2012; Thibault et al., 2005; Vogt & Schramm, 1991). Critically, these declines have led to significant and persistent ecosystem‐wide impacts (Bianchelli, Buschi, Danovaro, & Pusceddu, 2016; Wernberg, Bennett, et al., 2016; Wikström & Kautsky, 2007). There is currently a lack of understanding of the long‐term ecological implications of these changes and how such ecosystem changes might be reversed.

Although Laminariales appear to respond well to passive forms of conservation (e.g., implementation of MPAs where top down control prevails), the extent to which these strategies confer similar benefit to fucoids is unknown and integrating a variety of approaches is likely required. Understanding the specific response of fucoids to stressors and the mechanisms facilitating or hindering recovery will be key for designing appropriate and informed management strategies. Furthermore, emerging cutting‐edge anticipatory techniques such as assisted adaptation or evolution (Aitken & Whitlock, 2013; van Oppen, Oliver, Putnam, & Gates, 2015) should be investigated as potential avenues to boost resilience of existing populations to change. Addressing the dearth of information on subtidal fucoid community ecology, particularly in Australia where fucoids dominate canopy diversity of reefs, will be critical for managing subtidal reefs into the future.

## CONFLICT OF INTEREST

None declared.

## AUTHOR CONTRIBUTIONS

MAC and TW each contributed equally to all aspects of the concept, research, interpretation, and writing of this manuscript.
