# Supplementary material for: Forgotten underwater forests: The key role of fucoids on Australian temperate reefs
Source: Ecol Evol. 2017 Sep 10;7(20):8406–18. doi: 10.1002/ece3.3279 (PMC5648665; doi:10.1002/ece3.3279)
Supplement: Supplementary file 1 [file ECE3-7-8406-s001.docx]

**S1 Appendix**

**Protocol for literature search**

A literature search was carried out using the Web of Science Core Collection on 12/4/17 to assess the number of publications on *Phyllospora comosa*, *Scytothalia dorycarpa, Durvillaea potatorum, Acrocarpia, Cystophora (grevellei, monilifera, moniiformis, pectinata or racemosa), Cystoseira trinodis*, *Sargassum* spp., *Macrocystis pyrifera* and *angustifolia, Lessonia corrugata and* *Ecklonia radiata.* These species names were searched for separately in the title, keywords or abstract of papers without any restrictions. Given the sheer volume of papers on *M. pyrifera* due to its global distribution, and on *Sargassum* due to its species diversity and abundance on both tropical and temperate reefs, we also limited our search for these species to papers from Australia prior to identifying those relevant to our study (see Table S1). This search was conducted by one researcher (Melinda A. Coleman) to avoid bias in selecting publications for inclusion in the study following the search.

Irrelevant publications were omitted if (a) they mentioned one of the species in the search criteria but were about another taxa (e.g. a herbivore that consumes the target taxa) and did not provide any relevant information on the species of interest, (b) were erroneous publications that had nothing to do with any of these taxa (c) were focused on taxonomy or phylogeography and not relevant for the purposes of this study or (d) focused on chemical properties and their application or (e) were conducted in another country or on tropical or invasive species (e.g. many papers on *Sargassum*). Following omission of irrelevant papers, we compiled a list of between 1 and 25 papers for fucoids and 311 and 44 papers for *Ecklonia radiata and Macrocystis*. Given that many of the fucoids have an exclusively Australian distribution, we further refined our search to omit papers on *Ecklonia radiata* if they were conducted on this species outside of Australia (however, both sets of data are presented because any study on *Ecklonia radiata* contributes to our broad understanding of this species). Of the 311 *Ecklonia radiata* publications, 234 were from Australia and are directly comparable to the other species.

Given the lack of studies on fucoids a rigorous quantitative metanalysis of the relative importance of these species compared to co-occurring laminariales was not feasible. Further, many early publications were descriptive and data could not be extracted to test hypotheses about the relative importance of fucoids relative to laminariales. Hence, we used the identified publications to conduct a qualitative review of knowledge on these fucoids, with a focus on those that we know more about (*Phyllospora comosa*, *Scytothallia dorycarpa* and *Sargassum* spp.), to demonstrate their importance and identify knowledge gaps. The few publications and lack of qualitative data on these fucoids highlights the main message of our manuscript that there exists a dearth of information relative to co-occurring laminarian taxa and that this knowledge gap should be addressed in order better understand these key habitats and inform conservation and rehabilitation efforts.

Table S1. Summary of search statistics for each species. * = given the large volume of papers we modified our search to limit papers to Australia prior to searching for papers that were relevant to this study.

|  |  | **Total papers** | **Relevant papers** |
| --- | --- | --- | --- |
| ***Fucales*** | ***Phyllospora comosa*** | 29 | 25 |
|  | ***Scytothalia dorycarpa*** | 8 | 8 |
|  | ***Durvillaea potatorum*** | 32 | 5 |
|  | ***Sargassum spp.*** | 112* | 22 |
|  | ***Acrocarpia spp.*** | 15 | 3 |
|  | ***Cystophora (5 spp.)*** | 13 | 1 |
|  | ***Cystoseira trinodis*** | 10 | 1 |
| ***Laminariales*** | ***Macrocystis spp.*** | 44* | 10 |
|  | ***Ecklonia radiata*** | 394 | 311  (234 Australian) |
|  | ***Lessonia corrugata*** | 8 | 0 |
